# Paraquat induces oxidative stress, neuronal loss in substantia nigra region and Parkinsonism in adult rats: Neuroprotection and amelioration of symptoms by water-soluble formulation of Coenzyme Q_10_

**DOI:** 10.1186/1471-2202-10-88

**Published:** 2009-07-27

**Authors:** Mallika Somayajulu-Niţu, Jagdeep K Sandhu, Jerome Cohen, Marianna Sikorska, TS Sridhar, Anca Matei, Henryk Borowy-Borowski, Siyaram Pandey

**Affiliations:** 1 Chemistry & Biochemistry, University of Windsor, Windsor, ON, Canada; 2 Psychology, University of Windsor, Windsor, ON, Canada; 3 Institute for Biological Sciences, National Research Council Canada, Ottawa, ON, Canada; 4 St John's Research Institute, Bangalore, India

## Abstract

**Background:**

Parkinson's disease, for which currently there is no cure, develops as a result of progressive loss of dopamine neurons in the brain; thus, identification of any potential therapeutic intervention for disease management is of a great importance.

**Results:**

Here we report that prophylactic application of water-soluble formulation of coenzyme Q_10 _could effectively offset the effects of environmental neurotoxin paraquat, believed to be a contributing factor in the development of familial PD. In this study we utilized a model of paraquat-induced dopaminergic neurodegeneration in adult rats that received three weekly intra-peritoneal injections of the herbicide paraquat. Histological and biochemical analyses of rat brains revealed increased levels of oxidative stress markers and a loss of approximately 65% of dopamine neurons in the *substantia nigra *region. The paraquat-exposed rats also displayed impaired balancing skills on a slowly rotating drum (rotorod) evidenced by their reduced spontaneity in gait performance. In contrast, paraquat exposed rats receiving a water-soluble formulation of coenzyme Q_10 _in their drinking water prior to and during the paraquat treatment neither developed neurodegeneration nor reduced rotorod performance and were indistinguishable from the control paraquat-untreated rats.

**Conclusion:**

Our data confirmed that paraquat-induced neurotoxicity represents a convenient rat model of Parkinsonian neurodegeneration suitable for mechanistic and neuroprotective studies. This is the first preclinical evaluation of a water-soluble coenzyme Q_10 _formulation showing the evidence of prophylactic neuroprotection at clinically relevant doses.

## Background

Parkinson's disease (PD), one of the common neurodegenerative disorders, results from progressive loss of dopamine (DA) neurons in the *substantia nigra pars compacta *(SNpc). More than 95% of PD cases are sporadic and the disease often begins after the age of 60 years [1,2]. Symptoms, such as resting tremors, postural instability, rigidity and bradykinesia, become marked upon the loss of approximately 80% of the DA neurons. Along with these motor deficits, PD patients exhibit loss of balance that may be implicated in their impaired ability to voluntarily change gait stride or direction and their psychological fear of falling [3].

Although the exact causes of sporadic PD remain unclear, numerous environmental risk factors, especially neurotoxins, have been implicated in its etiology [4]. Environmental toxins such as rotenone, maneb (MB) and paraquat (PQ, 1,1-dimethyl-4,4-bipyridinium) have been shown to induce PD-like symptoms in experimental animals [5]. A recent study of an East Texas population that used a case-control design revealed an elevated risk of PD from exposure to organic pesticides such as rotenone and paraquat [6]. Experimental evidence of the neurotoxicty of such organic compounds has been demonstrated in mice and rats. Exposure to PQ alone or in combination with MB in such animals has been shown to result in loss of DA neurons in SNpc and to reduce the animals' general activity [7-10].

The chemical structure of PQ is similar to the known dopaminergic neurotoxin, N-methyl-4-phenylpridinium ion (MPP^+^), the active metabolite of MPTP. Thus, it belongs to the class of redox cycling compounds capable of inducing mitochondrial damage, increase ROS production and oxidative stress [11,12]. Ultimately, it may be important to develop an animal model of PD that consists of sensitive behavioural indicators of early onset of DA neurodegeneration in the SNpc and to test the effectiveness of any neuroprotective agents. Obviously application of such a substance during the early stage of PD in humans could help them maintain a normal quality of life. Rather than examining the effects of any neuro-protective agent on maintenance of animals' general activity, one should investigate its effects on more sensitive behavioral indicators such as balance and gait performance. Wishaw et al. (2003) devised a rotorod test for examining impaired gait performance of rats exposed to near complete unilateral destruction of the substania nigra [13]. They found that such rats exhibited contra-lateral inflexible digit extension and a general hunched posture when forced to walk on a slowly rotating (12 R.P.M.) drum ("rotorod"). Less severe but more general destruction of the substantia nigra in these animals might produce less dramatic and more specific effects due to reduced balance. That is, such animals would be expected to show less spontaneous variability in maintaining their balance on the rotorod.

Oxidative stress burden in the midbrain is usually high even under normal conditions, due to generation of reactive metabolites of DA, and is further elevated in PD patients [11]. Currently there is no cure for PD, however, there is an ongoing search for reliable neuroprotectants that could diminish the rate of neurodegeneration and help patients maintain a better quality of life. Thus, agents capable of improving the mitochondrial function and inhibiting oxidative stress should provide neuroprotection [14]. To this effect, antioxidants such as vitamin E and Coenzyme Q_10 _(CoQ_10_) have been extensively evaluated in both preclinical animal studies and clinical trials [15-18]. CoQ_10_, especially, continues to attract attention as it is not only a critical component of the mitochondrial respiratory chain complexes, it is also a powerful antioxidant [19]. Its clinical applications, however, show a low efficacy, probably because of its lack of solubility in aqueous media and poor bioavailability. A recently developed water soluble formulation of CoQ_10 _(WS-CoQ_10_) addresses these issues to offer potentially greater benefits and a wider range of applications [20]. This WS-CoQ_10 _formulation protects neuronal cells *in vitro *from glutamate excitotoxicity [21] and direct oxidative stress [22]. It is also effective in mitigating the brain damage caused by ischemia in experimental rats [23].

Previously, we have shown that PQ causes mitochondrial dysfunction and apoptosis in differentiated human neuroblastoma cells and that pretreatment of cells with WS-CoQ_10 _offers neuroprotection [24]. Here we describe a model of PQ-induced neurodegeneration in adult Long Evans hooded male rats and the application of WS-CoQ_10 _in drinking water to offset such toxicity. Using histological, biochemical and behavioural assessments, we established that systemic exposure of rats to PQ caused oxidative stress, loss of DA neurons in SNpc, and affected the animals' motor skills, as evidenced by a reduced variability in balancing on a rotorod. More importantly, we found neuroprotection and amelioration of these PD-like behavioral symptoms in rats given WS-CoQ_10 _in their drinking water.

## Methods

### Water-soluble formulation of CoQ_10 _(WS-CoQ_10_)

Water-soluble formulation was prepared from CoQ_10 _(Kyowa Hakko, New York, NY) and polyoxyethanyl α-tocopheryl sebacate (PTS) by directly combining both components in a molar ratio of 1: 2 mol/mol (1: 3 w/w) and heating them to a temperature higher than their respective melting points to form a clear melt, which is water-soluble and can be diluted with aqueous solutions (e.g., water, saline, phosphate buffered saline) to a desired concentration. PTS was synthesized by conjugating polyethylene glycol 600 to α-tocopherol via bi-functional sebacic acid (Sigma-Aldrich, St. Louis, MO) as previously described [20]; .

Typically, stock solutions of WS-CoQ_10 _(50 mg of CoQ_10 _and 150 mg of PTS per ml in PBS (phosphate buffered saline) or a placebo (150 mg of PTS per ml of PBS) were made and stored at 4°C. They were subsequently diluted with regular drinking water to a final concentration of 50 μg CoQ_10 _and 150 μg PTS per ml (WS-CoQ_10_) or 150 μg PTS per ml (placebo).

#### Experimental animals and their general care

**Figure 1:**
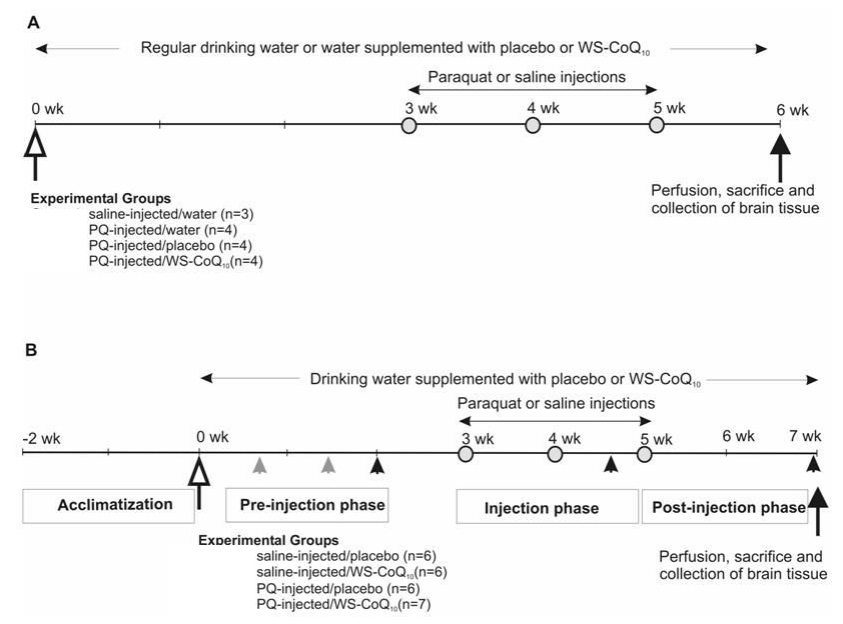
**Schematic outlines of experimental procedures**. A) Experiment 1: Four experimental groups of animals were provided with indicated water or water supplemented with placebo or WS-CoQ_10_. Three weeks later, they received intraperitoneal injections (open circles) of saline or PQ (10 mg/kg) once a week for 3 consecutive weeks. One week following the last injection (dark arrow), rats were sacrificed and brain tissue was collected for immunohistochemical studies. B) Experiment 2: Four experimental groups of animals were given either placebo or WS-CoQ_10 _supplemented drinking water (as indicated). Three weeks later, they received intraperitoneal injections (open circles) of saline or PQ (10 mg/kg) once a week for 3 consecutive weeks. Rats were trained on the rotorod during the pre-injection period (grey arrowheads) and behavioural assessment was conducted on the third, fourth and fifth rotorod sessions (dark arrowhead). Two weeks following the last injections, rats were sacrificed and brain tissue was collected for biochemical studies (dark arrow).

All animal care, treatments, and procedures were approved by the University of Windsor's Animal Care Committee in accordance with the Canadian Council for Animal Care guidelines. A total of forty-seven adult male Long-Evans hooded rats purchased from Charles River Laboratories (St. Canstant Quebec). The rats were approximately 3.0 months old at the start of the experiments. Rats were housed in group cages (3–4 rats per cage) daily except during 2 hours afternoon feeding times, in accordance with Canadian Government animal care guidelines for long-term maintenance. In view of this requirement, rats in each cage had to receive the same specific drinking water regimen and injection condition in order to separate saline-injected from paraquat injected animals. This was necessary to prevent the development of any dominance hierarchies based on degree of neurodegeneration. Otherwise, saline-injected or other neuroprotected rats might become dominant over their cage mates subjected to neurodegeneration and thus gain more access to food and liquids. However, it is important to note that group caging of rats with the same liquid and injection conditions, is not sufficient a guarantee against the development of dominance hierarchies that is normal in such social animals. Thus the possibility would remain that less dominant animals might not gain equal nutritional access. In order to avoid this, we also instituted a separate 2 hour feeding schedule every afternoon, where each animal was isolated in an individual holding cage and provided 25 g of dry rat chow and its specific liquid treatment. These maintenance procedures insured that rats consumed enough food to insure normal weight gain over the course of the experiment from their starting weights of 300–320 g to 410–450 g by the end of the experiment. This feeding schedule also promoted drinking further insuring that each rat received its normal daily amount of liquid. Drinking solutions (regular water, water supplemented with WS-CoQ_10 _or with the placebo carrier solution) were always available in group and individual holding cages but food was only available in the individual cages. Water bottles containing WS-CoQ_10 _and placebo were covered with aluminium foil and all bottles were refilled every three days. The animal colony room was maintained on a reversed 12 h:12 h light: dark cycle with the temperature maintained at 20°C. Our study consisted of two separate experiments (figure 1A–B) described as follows.

#### Experiment 1

This experiment was designed to determine whether the original paraquat (PQ) injection protocol used with mice [7] could produce similar neurodegenerative effects in Long-Evans hooded rats, and if so, whether WS-CoQ_10 _supplement in their drinking water could offer any protection from the toxic effects of paraquat. This experiment initially contained twenty naïve adult male Long-Evans hooded rats, partitioned into four separate groups, of which two received plain drinking water, one received drinking water supplemented with WS-CoQ_10_, and the other received drinking water supplemented with the carrier solution (Placebo) throughout the course of the experiment (Figure 1A). Since placebo (also called PTS in our study) is a pro-drug form of vitamin E (in which tocopherol was chemically derivatised by sebacic acid and PEG) and is used as a component (carrier) in WSCoQ_10 _formulation [23], the possible neuroprotective effects of CoQ_10 _were compared to placebo and water groups. After two weeks from the beginning of their respective drinking water treatments, rats were given 3 weekly intraperitoneal injections of saline or paraquat (10 mg/kg body weight dissolved in PBS). One group that received plain drinking water was injected with saline (Saline-Water group). The other three groups were injected with paraquat (PQ-Water group; PQ-Placebo group; PQ-CoQ_10 _group). One week after their last injection, rats were perfused with heparinised tyrode's solution (pH 7.4) followed by 10% neural buffered formalin and sacrificed. Brains were removed and fixed in 10% buffered formalin for another 12 h and embedded in paraffin for histochemical analysis. We note that technical difficulties prevented accurate assessment of TH-positive neurons in the Saline-Water group. Therefore, we added two more age-matched animals from the same colony to this group for further assessment.

#### Experiment 2

This experiment was designed not only to conduct histological/biochemical assays of PQ-induced neurodegeneration, but also to analyze a potential PD-like behavioural changes resulting from such exposure (Figure 1B). In addition, we intended to determine whether WS-CoQ_10 _treatment could offset PQ-induced behavioural impairment. In view of the fact that no differences occurred between the PQ-Placebo and PQ-Water treatment groups in Experiment 1, we eliminated plain water treatment, but gave animals either WS-CoQ_10 _or its carrier solution PTS (Placebo) in their drinking water. We used twenty-five naïve adult male Long-Evans hooded rats for this experiment and exposed them to the same basic injection schedule as in Experiment 1. Twelve animals always received drinking water supplemented with the WS-CoQ_10 _and were divided into two equal injection groups (Saline-CoQ_10 _and PQ-CoQ_10_). The other 13 rats always received the placebo supplemented drinking water and were also divided into two groups based on the injection regime, resulting in the Saline-Placebo group (6 animals) and the PQ-Placebo group (7 animals). Rats were sacrificed two weeks after the last injection, as opposed to one week after the last injection in experiment 1. This added week allowed us to carry out post-injection behavioural assessments of gait performance on the rotorod.

### Behavioural assessment

#### Rotorod Apparatus

We measured rats' ability to maintain their balance on a rotorod as per a previously described protocol [13]. This rotorod apparatus consisted of a paper-covered (80 grid) 15 cm long, 7 cm diameter wooden dowel attached to a variable speed motor. Clockwise revolutions of the rotorod could be adjusted from 6 to 12 R.P.M. The rod was separated from the motor by a vertical 30 × 48 cm grey wooden panel that was scored with black vertical and horizontal lines to form 12 × 12 cm squares as shown in Figure 4B. The apparatus rested on a small table so that the dowel was 27 cm above its surface. A digital video camera (Orbyx Electronics Inc, CA) was positioned 1 m in front of and level with the rod. The apparatus was illuminated by regular fluorescent ceiling lighting and a 60-W lamp approximately 3 m in front of it.

#### Experimental procedure

Experiment 2 setup consisted of a two week pre-injection phase followed by an injection phase of three weeks and a two-week post-injection phase. Rats received three rotorod sessions during the pre-injection phase, spaced approximately two days apart over the two-week period, one rotorod session five days after the second injection (mid-injection test), and one session in the post-injection phase given one week after their last injection. The first two pre-injection sessions served to train the rats to maintain their balance on the rotorod while we gradually increased its rotation speed from 6 to 12 R.P.M. From the third test on, we maintained the rotorod's rotation speed at 12 R.P.M. Rats' performance on the third pre-injection test provided baseline performance for comparison with the next two tests. Rats were always placed in the forward position (facing left) as the rod rotated clock-wise.

#### Dependent Behavioural Measures and Statistical Analyses

Each rat's video recording was converted to 5 frames per second still pictures from which we recorded its movements over the last 100 seconds (500 frames) with a software tracking program (7 Software, Inc, Montana, U.S.A.). Figure 4B illustrates a picture frame superimposed on the tracking frame composed of 450 vertical and 450 horizontal pixels. It was noted that although rats were initially oriented to walk forward on the slowly rotating rotorod, they also turned around and walked backward during some portions of these sessions. Therefore, we calculated the amount of time each rat actually spent (proportion of frames) walking backward as well as its average horizontal and vertical nose locations as it walked in each direction.

Each rat's proportion of time spent walking backward was transformed into an arcsin 2√X measure to enhance homogeneity of variance for parametric statistical analysis of proportion scores [25]. Each dependent measure was analyzed by 2 (Injection: PQ vs. Saline) by 2 (Liquid Supplement: CoQ_10 _vs. Placebo) by 3 (Rotorod Test Session: pre-, mid-, post-injection) analysis of variance (ANOVA) with repeated measures on the last factor. We conducted statistical analyses of the behavioural data with SPSS (version 17) program and report significant effects at *p *< .05.

#### Immunohistochemistry

Rats from experiment 1 (Figure 1A) were perfused with heparinised tyrode's solution (pH 7.4) followed by 10% neural buffered formalin and sacrificed. Brains were removed and fixed in 10% neural buffered formalin for another 12 h and embedded in paraffin. Coronal sections (8 μm) were cut across the whole substantia nigra region and processed for immunohistochemistry using a stereotaxic rat brain atlas. Briefly, sections were deparaffinized and incubated with Tris-buffered saline (TBS: 50 mM Tris-HCl, 150 mM NaCl, pH 7.6) containing 3% H_2_O_2 _for 10 min at room temperature to destroy endogenous peroxidise activity. Nonspecific IgGs were blocked with universal blocking solution (DAKO diagnostics Canada Inc., Mississauga) for 30 min at room temperature. Excess liquid was drained, and sections were incubated in a humid chamber with a rabbit polyclonal anti-tyrosine hydroxylase antibody (1:1000 Pel-Freeze Biologicals, USA) for 24 h at 4°C. Negative controls included the omission of primary or secondary antibodies. Sections were then washed in TBS and incubated with DAKO Envision peroxidase conjugated to goat anti-rabbit IgG for 30 minutes at room temperature. Immunolabelling was detected using 0.02% diaminobenzidine tetrachloride and 0.006% hydrogen peroxide for 10 min at room temperature. Sections were washed in running water and then counterstained with Mayer's hematoxylin. The images were captured using a Carl Zeiss Axiovert 200 M microscope. The loss of dopaminergic neurons was determined by counting TH-immunopositive cells (cell body) under bright-field illumination. After delineation of the SN pars compacta using the 10H objective, every 5^th ^section from the entire substantia nigra region (a total of 15–20 slides) was counted using 20H objective and the average of all counts represented the total number of neurons per section. Multiple sections were counted to obtain the TH+ve neuronal numbers. The entire region from the beginning to end was scanned.

#### Tissue fractionation

In both experiments rats designated for biochemical assays were sacrificed by CO_2 _inhalation and their brains were removed. These isolated brains were washed with ice-cold PBS and placed on ice until further dissection. Midbrain region was dissected, weighed and homogenized in an ice-cold isolation medium (50 mM Tris-HCl, 150 mM NaCl, 1 mM EDTA, 1 mM PMSF, 1 mM benzamidine, 10 μg/ml pepstatin A and 10 μg/ml leupeptin; 5 ml buffer/1 g tissue). The homogenates were centrifuged at 500 × g for 10 min. The pellets were discarded and the supernatant was used as the cytosolic fraction in all subsequent assays. Protein content was measured using the BioRad assay.

#### Glutathione assay

Three rats from each group in Experiment 2 were designated for the GSH assay. This assay was performed essentially as previously described [26]. Briefly, 50 μl of cytosolic fraction (obtained from the midbrain) and 100 μl of reaction mixture (1 mM NADPH, 100 mM Na_2_HPO_4_, 100 units GSH reductase and 1 mM DTNB) were incubated for 15 min at 37°C in a 96 well plate. Absorbance was measured at 412 nm using a Bio-tek ELx 808 ru Ultra Micro Plate Reader. GSH levels were determined from the standard curve constructed using 1 mM GSH. Levels of GSH were expressed per microgram protein.

#### Lipid Peroxidation Assay

Two rats from each group in Experiment 1 were designated for the lipid peroxidation assay. It was performed as previously described [27]. Briefly, 100 μl of the cytosolic fraction of the midbrain was added to 1 ml of thiobarbituric acid and incubated at 100°C on a heat block for 20 min. After cooling the tubes, absorbance was measured at 535 nm (Genesys spectrophotometer). Lipid peroxidation levels were determined from the standard curve prepared using 1 mM malondialdehyde (MDA). Lipid peroxidation levels were expressed per microgram protein.

### Statistical analysis

Unless otherwise stated, all the biochemical experiments have been performed in triplicates in 3 animals, but TH counting was performed by the counting 10 slides for each animal and three separate animals were used for counting. One-way analysis of variance (ANOVA) followed by *post-hoc *Bonferroni's multiple comparisons test was used to assess statistical differences. Significant effects at *P *< 0.05 are reported. Statistical tests were performed using GraphPad Prism version 4.0.

## Results

### The effects of PQ and WS-CoQ_10 _on DA neurons in the midbrain

**Figure 2:**
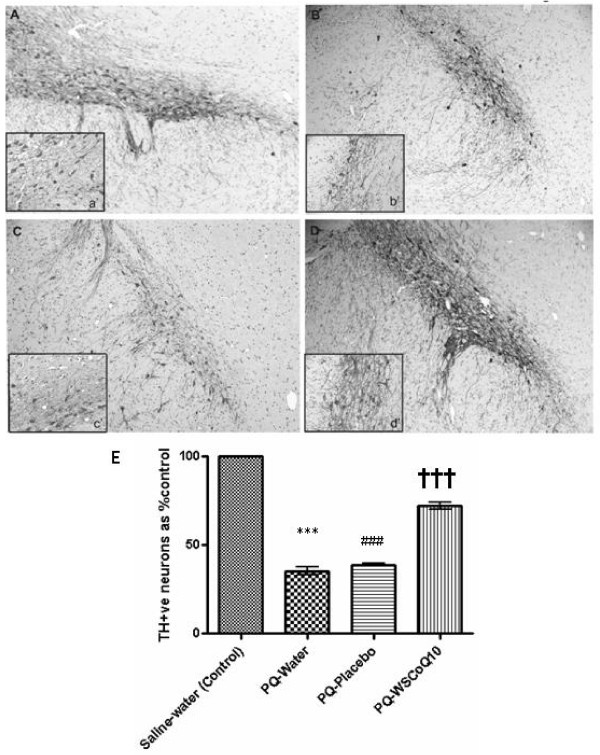
**Immunohistochemical evaluation of midbrain damage**. Experiment1: Immunohistochemistry performed using anti-tyrosine antibodies on the brain sections of animals. Representative photomicrographs of midbrain sections showing TH-immunopositive neurons from saline injected animals (A), paraquat injected water fed animals (B), paraquat injected placebo fed animals (C) and paraquat injected WS-CoQ_10 _fed animals (D). Magnification: 10H. Insets (a' to d') show the density of TH-immunopositive neurons and fibres in the respective groups. Magnification: 20×. (E) The number of TH-immunopositive neurons counted in every fifth section throughout the *substantia nigra pars compacta*. Data are shown as mean ± SEM. Note: Significant differences (*p *< .001) ***between saline injected-water fed group and and paraquat injected water fed group; ### between saline injected-water feds and paraquat injected-placebo fed group; ^HHH ^between paraquat injected WS-CoQ_10 _fed group and paraquat injected placebo fed group (evidence of neuroprotection).

Microscopic examination of midbrain sections revealed reduced TH immunostaining in the PQ-injected rats (both water and placebo-fed groups, Figure 2B and 2C, respectively) as compared to the control saline-injected rats (Figure 2A). There was also a diminished staining of TH-positive fibres, indicative of neuronal loss and reduced density of the fibres (compare Figure 2B-b' and 2C-c' to 2A-a'). These PQ-injected animals received either regular drinking water or placebo throughout the duration of the experiment. In striking contrast were the images of the PQ-WS-CoQ_10 _midbrain sections, which showed a strong TH immunostaining of both the cell bodies and the fibres (Figure 2D and 2D-d'). This group of rats was given the WS-CoQ_10 _supplemented drinking water, which might have counteracted the effects of PQ. The immunostaining data was confirmed by counting the TH -positive cells in the entire region of SNpc (Figure 2E). The TH -positive cells results were expressed as percent control. The counts revealed a nearly 65% reduction of the cell number in the PQ-injected rats receiving regular drinking water (from 313.7 ± 11.3 in Saline group to 110.33 ± 8.5 in PQ-placebo group, *P *< 0.001) and 60% reduction in the placebo fed group (down to 121.33 ± 2.081). The cell counts also confirmed the neuroprotective effects of WS-CoQ_10_. Although the estimated number of TH-positive neurons in PQ-WS-CoQ_10 _group was lower than in control saline group, their total number was still 2.5-fold higher than in PQ-water group (*P *< 0.001) and 1.5 times higher than in PQ-WS-placebo group (*P *< 0.001). Interestingly, the counts also revealed a higher number of TH-positive cells in PQ-Placebo group in comparison to PQ-Water group (*P *< 0.001). It should be noted here that the placebo solution contained PTS, a derivatised form of vitamin E, which could be responsible for this neuroprotection.

### Assessment of oxidative stress markers

**Figure 3:**
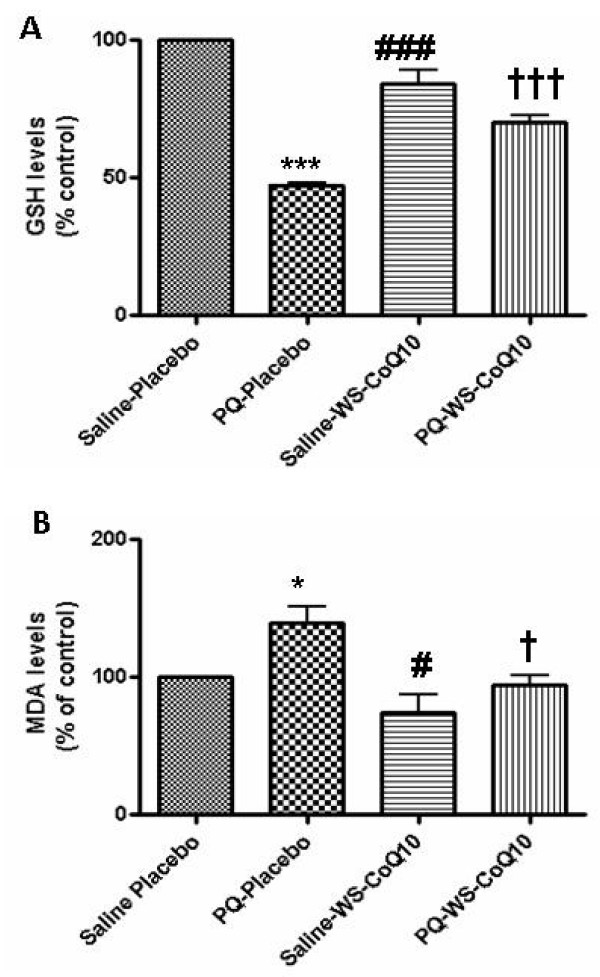
**Oxidative stress markers**. Experiment 2: Following behavioral testing, rats were sacrificed, midbrain tissue was homogenized and assayed for GSH (A) and MDA levels (B). Data (mean ± SEM) was normalized to that of saline injected placebo fed group, which was taken as 100%. (A) statistically significant differences (*p *< .001): *** between saline injected placebo fed group and paraquat injected placebo fed group; ^### ^between paraquat injected placebo fed group and saline injected WS-CoQ_10 _fed group; ^HHH ^between paraquat injected placebo fed group and paraquat injected WS-CoQ_10 _fed group. (B) Statistically significant differences (*p *< .05): * between saline injected placebo fed group and paraquat injected placebo fed group; ^# ^between paraquat injected placebo fed group and saline injected WS-CoQ_10 _fed group; ^H ^between paraquat injected placebo fed group and paraquat injected WS-CoQ_10 _fed group.

We measured the GSH content (Figure 3A) and lipid peroxidation (Figure 3B) in the midbrain of experimental animals as the markers of oxidative stress. We compared the levels of these markers in the brains of PQ-injected groups (Placebo and WS-CoQ_10 _fed groups) with the saline injected groups (Placebo and WS-CoQ_10 _fed groups).

GSH is an abundant antioxidant in many tissues, including the brain and is known to protect cells against oxidative damage [28,29]. Depletion in GSH levels has been observed in the MPTP model of PD [30]. In this study, the GSH content in the PQ-placebo group was reduced by half as compared with the saline groups (*P *< 0.001), consistent with it being utilized to combat the effects of PQ (Figure 3A). However, the GSH level was maintained at nearly control level in the PQ-WS-CoQ_10 _group (Comparing both the paraquat injected groups, *P *< 0.001), suggesting that the antioxidant content of WS-CoQ_10 _formulation offsets the effect of PQ, sparing the GSH system.

Recent studies report a higher malondialdehyde (MDA) level [31] and increased lipid peroxidation in the substantia nigra region of PD-exposed brains [32]. Here we measured the content of MDA, the product of lipid peroxidation, to assess the degree of PQ-induced lipid damage (Figure 3B). The data showed that the damage was particularly evident in PQ-water group as indicated by a significantly increased MDA level in comparison to groups injected with paraquat and fed with either placebo or WS-CoQ_10 _(*P *< 0.05). Again, the MDA level in PQ-WS-CoQ_10 _was nearly the same as in the saline injected groups and it was significantly lower than in the PQ-placebo group (*P *< 0.05). Thus, these results indicated that PQ injections lead to the increased lipid peroxidation, which was mitigated by WS-CoQ_10_.

In summary the biochemical and histological assessments indicated that PQ injections induced oxidative stress in the brain leading to lipid peroxidation, reduction of GSH and 70% loss of TH positive neurons in the SNpc region. The histological and biochemical assessments also revealed that the toxicity of PQ towards DA neurons was neutralized by the WS-CoQ_10_formulation.

### Behavioural Assessments

**Figure 4:**
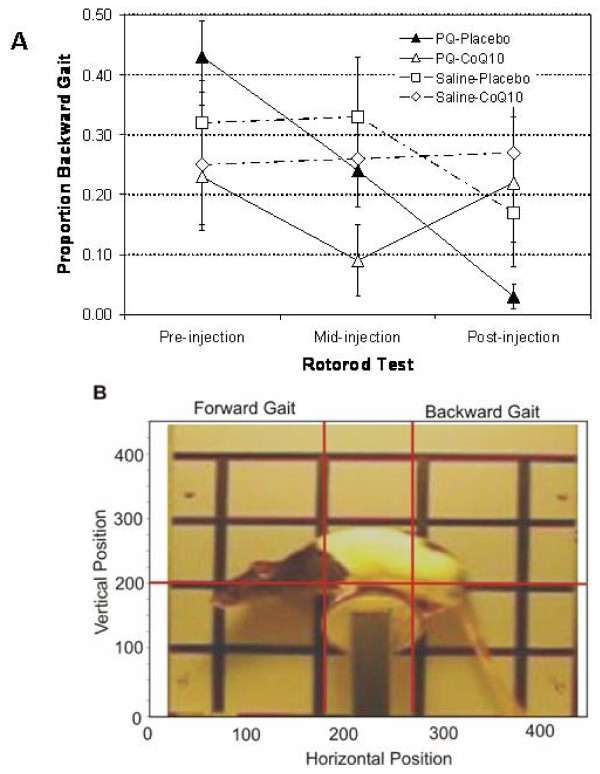
**Behavioural Assessments**. Rats were trained on the rotorod and behavioural testing was carried out. A) Rats were placed on a moving rotorod and the time spent by the rats walking backwards on the rotorod was monitored. Measurements were made before (pre-injection), during (mid-injection) and after (post-injections) the PQ-injections. The data points represent the mean proportion of time spent walking backwards during each testing session B). Representative image of the tracking format used for determining forward/backward walking posture as well as the location of nose on the rotorod. The horizontal line is positioned at the top of the rotorod and the two vertical lines determine the forward and backward walking positions. Any nose locations between the left and right vertical lines were not counted.

As already noted, we subjected rats to a series of rotorod tests over the course of Experiment 2 to measure spontaneous changes in their balancing behaviour. These neuro-protective effects were accompanied by maintenance or even improved balance on the rotorod (Figure 4A). In fact, animals of the PQ injected WS-CoQ_10 _group behaved the same on the rotorod tests as animals in either saline injected groups.

### Proportion of Time Spent in Walking Backward

Figure 4A shows the changes in the amount of time rats in each group spent walking backward over the three test sessions (pre-injection, mid-injection, post-injection). Although all groups spent a greater proportion of their time walking forward, they spent almost 30% to 40% of their time walking backwards on their first (pre-injection) test. Only rats in the PQ-Placebo group steadily reduced the amount of time spent spontaneously walking backward. Therefore during the post-injection test, they almost exclusively maintained a forward walking gait. Rats in the PQ-CoQ_10 _group showed an increased time spent walking backward on the post-injection test as compared to those in the PQ-Placebo group. The rats in the Saline-Placebo group developed only slight declines, while those in the Saline-CoQ_10 _group showed little if any change. The three-way ANOVA on the transformed data uncovered a significant injection type among all three factors, *F *(2, 42) = 4.68, p = 0.015. One-way within-subjects ANOVAs for each group revealed that only rats in the PQ-Placebo group significantly reduced their proportion of walking backwards, *F *(2, 12) = 12.08. *p *< 0.01. There were no significant effects for any changes in the other three groups.

### Vertical and horizontal nose positions

Separate analyses of rats' vertical and horizontal nose positions did not uncover any significant changes reflecting the experimental treatments (i.e., PQ vs. saline injections or placebo vs. CoQ_10 _supplementation). As shown in Figure 4B, rats tended to walk forward with their noses slightly below the top of the rotorod (below the horizontal line at 210 pixels) and extended well away from it (177 pixels from the left vertical line). When rats did walk backwards, they also tended to extend their noses similarly slightly below the top of and away from the rotorod.

In summary, we found clear evidence of a motor balance dysfunction in rats that had suffered paraquat-induced neuronal loss in SNpc (PQ-Placebo group). These rats drastically reduced their tendency to spontaneously turn around and walk backwards during and following their series of injections. On the other hand, paraquat-injected rats whose SNpc area was protected by WS-CoQ_10 _(PQ-CoQ_10 _group) retained their pre-injection levels of the backward gait on the final post-injection. Thus this group did not differ statistically from either of the saline-injected groups.

## Discussion

Accumulated evidence suggests that PD may arise from a combination of genetic susceptibility and exposure to environmental toxins [32,4,33]. Indeed, several environmental risk factors such as metals, solvents, carbon monoxide and herbicides have been linked to the incidence and progression of PD [34-37]. Amongst these factors, the herbicide paraquat (PQ) shows clear neurotoxicity in the central nervous system. PQ can enter the CNS through neutral amino acid transporters associated with the blood brain barrier system [38] and selectively damage the SNpc neurons in mouse models [39]. It has also been reported that a prolonged exposure to non-pneumotoxic levels of PQ causes the damage to basal ganglia [33]. According to the published data, a range of xenobiotics, including paraquat and menadione, can undergo monoelectronic redox cycling in isolated brain capillaries, giving a rise to increased production of ROS [40,41]. Menadione, for example, is shown to significantly increase the permeability of endothelial cell monolayers *in vitro *[41]. Thus, there is a correlation between free radical formation and passive permeability of the cerebral microvascular endothelium; therefore, it is possible that the neurotoxicity of PQ may, in part, be linked to its ability to alter the permeability of the blood brain barrier.

Our data clearly confirmed the toxicity of systemically administered PQ, especially towards DA neurons of the SNpc. Three weekly intraperitoneal injections of this herbicide effectively killed between 50–70% of DA neurons in the midbrain of adult male Long-Evans hooded rats and induced PD-like symptoms. The effectiveness of PQ in killing DA neurons seems to vary from 40–70% in reported experimental studies, but its selectivity towards TH-positive neurons in the SNpc region is consitent in all studies. Ossowska *et al*., (2005) showed that long term paraquat administration caused somewhat less progressive neuronal loss of about 37% loss of dopamine neurons in adult male Wistar rats [42]. McCormack et al., (2002) found only about a 25% reduction in these DA neurons in C57BL/6 mice [32]. However, Brooks *et al*. (1999) found much greater losses of TH-positive neurons in the substantia nigra pars compacta (~60%) and their terminals in the striatum (~90%) after PQ exposure in C57BL/6J mice given the same dosage used in our study [7]. In the present study, we observed a loss of ~65% dopaminergic neurons using a 3 injection PQ regime. Perhaps, among other possible factors, the different strains of mice and rats used in these two sets of studies may account for the differences in susceptibility to PQ-induced neuronal loss.

The question remains whether our rats may have simply been susceptible to a more general, non-specific neurodegenerative effect from PQ exposure. Unfortunately, we did not test for this possibility by examining other brain areas. In view of this problem, we now use a general marker for neurons namely NeuN to stain various areas of brain in on-going experiments. Although we have yet to complete this study, our preliminary results from those rats that have completed it, clearly show a clear decrease in NueN-positive cell numbers in the midbrain area containing the SNc region but no decline in NeuN immunostaining in other areas of the brain; e.g. hippocampal region (data not shown) following PQ injections. Therefore we are confident that PQ exposure targeted DA neurons of the SNpc.

Moreover, the affected rats showed clear signs of deficiency in fine motor control as indicated by a reduced tendency to turn around and walk backwards on the rotorod. Furthermore, we have established that the brain damage and the performance deficits could be minimized if not prevented by giving the animals a water soluble formulation of CoQ_10 _(WS-CoQ_10_) throughout the experiment. This study is, in fact, the first pre-clinical evaluation of WS-CoQ_10 _as a neuroprotectant of DA neurons.

The neurotoxicity of PQ is linked to its ability to destabilize mitochondria and increase the production of reactive oxygen species (ROS) [12]. Experimental evidence indicates that PQ targets mitochondria of brain cells and the affected mitochondria are a major source of ROS. Using *in-vitro *assays, we show that PQ can destabilize the mitochondria isolated from mouse brains and from differentiated human neuroblastoma cells [24], (Somayajulu *et al.*, unpublished data). Furthermore ROS such as superoxide reacts with nitric oxide to form reactive nitrogen intermediates, which impair the mitochondrial respiratory chain and lead to decreased ATP synthesis [43]. Furthermore, we found evidence of increased lipid peroxidation, which could lead to changes in membrane properties and affect cellular homeostasis [33]. Therefore, the evidence suggests that dysfunctional mitochondria are most likely significant contributors to PQ-induced neurotoxicity.

Our data has also shown that placebo offers some protection against oxidative stress; however, placebo alone is not sufficient to protect the neurons from cell death induced by paraquat. The placebo formulation consists of Vitamin E and polyethylene glycol. Coenzyme Q_10 _on the other hand, works at the mitochondrial level and does prevents neuronal cell death induced by paraquat. Our data adds to the mounting evidence that antioxidants, especially CoQ_10 _and vitamin E, are important for the management of neurodegenerative diseases [16]. Neuroprotective effects of CoQ_10 _in the CNS have been extensively evaluated [18,44] and several in *vivo *studies demonstrate its protective role against experimental ischemia, sparing the levels of GSH and ATP [15,23]. CoQ_10 _is a highly hydrophobic, naturally occurring compound that mainly functions in mitochondrial membranes as a diffusible electron carrier for the mitochondrial respiratory chain complexes. It is also a powerful antioxidant readily scavenging free radicals [15]. Its pharmaceutical applications, however, seem to suffer from the lack of solubility and low bioavailability, both necessitating the applications of high doses to achieve a therapeutic effect. An open-label phase I clinical trial of CoQ_10 _in PD patients reveal a good absorption and tolerance of CoQ_10_, however, high dosages were required (up to 1200 mg per day) to achieve some beneficial effects [17,45]. A recent study by Cleren *et al *(2008) has shown that oil soluble Tishcon CoQ_10 _formula provided significant protection against MPTP toxicity in a mouse model [18]. However the effective doses used in this work were 200 mg/kg/day-1600 mg/kg/day (equivalent to 14–114 gm/day for a 70 kg patient). This dose is also extremely high and therefore unlikely to be used in human subjects. On the other hand, the effective daily dose of the water soluble formulation used in our study was 5 mg/kg/day in rats, roughly one fourth of the dose used in the cited above clinical trial and 40 times lower than the dose used by Cleren *et al *(2008) in mice [18]. The results from our study offer the possibility of using CoQ_10 _in smaller, clinically relevant doses.

The water soluble formulation of CoQ_10 _has been reported to be more efficacious as it combines two potent antioxidants, i.e., derivatised vitamin E (PTS) and CoQ_10 _[23,20], . PTS is a pro-drug form of vitamin E (alpha-tocopherol), which was chemically derivatised by sebacic acid and PEG and used as a component (carrier) in WSCoQ_10 _formulation [23]. These two compounds form a stable and water soluble complex, easy to deliver and test (i.e., in drinking water). The effective daily dose of this formulation that offered significant neuroprotection in our study will translate to 350 mg/day for a 70 kg human subject (roughly one fourth of the dose used in the aforementioned clinical trial). Indeed rats fed the WS-CoQ_10 _containing diet have shown elevated plasma levels of CoQ_10 _[23]. Due to the water-soluble nature of our formulation, we have done extensive work on its effect as neuroprotective agent in neuronal cell cultures [21,22,24]. In our previous *in vitro *studies we show that this formulation of CoQ_10 _protects differentiated SHSY-5Y cells against PQ toxicity by stabilizing mitochondrial membranes, maintaining mitochondrial membrane potential and sustaining ATP production [24]. More recently, we established that it inhibits Bax activity and prevents Bax-induced destabilization of mitochondria in mammalian cells [46].

Of equal importance in this study is the identification of the fine behavioural abnormalities (indicative of altered motor skills), observed in PQ-treated rats as the reduced behavioural variability for balanced walking on the rotorod (PQ-placebo group, Figure 4A). This was evidenced by rats reducing their spontaneous turning around to walk backward. These PQ-injected rats were receiving drinking water supplemented with derivatised vitamin E (PTS) as placebo and lost over 50% of the midbrain DA neurons (Figure 2E). It should be noted, however, that the rats of the PQ-Placebo group did not suffer extreme behavioural motor dysfunction of slow ambulatory behaviour with hunched postures, typically found in PD patients. Such PD-like general ambulatory behaviour has been observed in mice exposed to similar PQ and MPTP dosages [7] and a forward rotorod gaiting in rats receiving an unilateral destruction of the nigrostriatal bundle by targeted injections of 6-hydroxydopamine [13]. These behavioural differences, however, are not surprising given the differences in level of SNpc destruction in our animals compared with that of animals in the earlier studies.

Rats in the rotorod study described by Whishaw [13] obviously had most of their SNpc unilaterally destroyed which also reduced their flexibility in fore- and hind limb paw placement on the contralateral than ipsilateral side of the damage. Given the more general, but lower degree of neuronal loss in the PQ-Placebo group, it is not surprising that we observed the same flexibility of their paw placements as in the unaffected animals (both saline injected as well as PQ-WS-CoQ_10 _fed groups). It is noteworthy that symptoms of PD, such as resting tremors, postural instability, rigidity and bradykinesia, are not readily detectable until individuals have more than 80% loss of their SNpc neurons [11].

## Conclusion

We have identified subtle behavioural deficits that accompanied substantially low losses of DA neurons (approx. 65%) in the SNpc region. The most obvious implications of our results relate to the early detection of PD-like neurodegeneration. Reduced behavioural variability in the performance of simple motor or ambulatory tasks may signal the early onset of this disorder in humans. Given that CoQ_10 _or other neuroprotective agents can slow down or even stop further reduction of neuronal population, early detection and intervention could prevent further development of the symptoms. Further research will be required to establish the reliability and validity of such subtle behavioural diagnostic measures.

## Authors' contributions

All the authors contributed in the design of the experiments, manuscript preparation and approval for submission. MSN and SP contributed to most of the experiments including injection and feeding of different regiments, dissections, immunohistochemical analysis and biochemical analysis. JC, AM and VP were involved in the design and execution of behavioural tests, analysis of rotorod results and animal care. JS performed TH immunostaining and counting of the TH-positive neurons. MS and HBB prepared water-soluble CoQ10 and placebo formulations and CoQ_10 _measurements in tissues. TS was involved in experimental design, histo-pathological evaluations.
